# Mendelian randomization study shows no causal relationship between psychiatric disorders and glaucoma in European and East Asian populations

**DOI:** 10.3389/fgene.2024.1349860

**Published:** 2024-03-07

**Authors:** Yan Zhang, Longhui Fu, Fang Feng, Bo Liu, Ying Lei, Qianyan Kang

**Affiliations:** ^1^ Department of Ophthalmology, The First Affiliated Hospital of Xi’an Jiaotong University, Xi’an, China; ^2^ Department of Neurosurgery, The Second Affiliated Hospital of Xi’an Jiaotong University, Xi’an, China; ^3^ Department of Gynecology and Obstetrics, The First Affiliated Hospital of Xi’an Jiaotong University, Xi’an, China

**Keywords:** mendelian randomization, depression, insomnia, schizophrenia, glaucoma

## Abstract

**Background:** Glaucoma is a leading cause of blindness strongly associated with psychiatric disorders, but the causal association between glaucoma and psychiatric disorders remains uncertain because of the susceptibility of observational studies to confounding and reverse causation. This study aims to explore the potential causal association between glaucoma and three highly related psychiatric disorders (Depression, Insomnia, and Schizophrenia) in the European and East Asian populations using a two-sample Mendelian randomization analysis.

**Methods:** Instrumental variables (IVs) of depression, insomnia, and schizophrenia in the European population were obtained after strict filtering. Summary-level data for glaucoma and glaucoma subtypes (primary open-angle glaucoma and primary closed-angle glaucoma) were obtained as outcomes. The inverse variance weighting (IVW) method was used as the primary method. Additionally, the causal effect was evaluated in the East Asian population using the same methods to validate analysis results. The robustness of these results was confirmed using heterogeneity, pleiotropy, and Steiger directionality test.

**Results:** The primary MR results indicated that genetically driven psychiatric disorders were not causally associated with glaucoma (Depression: odds ratio (OR): 1.15, 95% confidence interval (CI): 0.93–1.42, *p* = 0.20; Insomnia: OR: 1.14, 95% CI: 0.63–2.05, *p* = 0.66; Schizophrenia: OR: 1.00, 95% CI: 0.93–1.08, *p* = 0.95), either with the risk of glaucoma subtypes in the European population. Meanwhile, results in the East Asian population were consistent with the results among the European population (Depression: OR = 1.38, CI 0.75–2.53, *p* = 0.30; Insomnia: OR = 0.99, CI 0.83–1.18, *p* = 0.93; Schizophrenia: OR = 1.06, CI 0.94–1.20, *p* = 0.34) with similar causal estimates in direction. Consistency was obtained by corroborating with other supporting methods. Besides, the robustness of the results was proved and the directionality test confirmed our estimation of potential causal direction (*p* < 0.001).

**Conclusion:** This study found a non-causal association between psychiatric disorders and the risk of glaucoma in the European and East Asian populations, which contradicts many existing observational reports, indicating that increased psychiatric disorders in glaucoma patients were more likely modifiable rather not inheritable.

## 1 Introduction

Glaucoma is a group of optic neuropathies characterized by progressive degeneration of retinal ganglion cells, resulting in visual field defects (Quigley, 2011). It has been estimated that the total number of patients would up to 111.8 million by 2040, making glaucoma the most common cause of irreversible blindness worldwide (Tham et al., 2014). Based on the anatomy of anterior chamber angle, glaucoma is categorized as open-angle glaucoma (OAG) and angle-closure glaucoma (ACG). Its pathogenesis is related to various genetic mutations and somatic diseases, indicating that glaucoma is a complicated genetic disorder (Wändell et al., 2022). Specifically, it was found that primary open-angle glaucoma (POAG) is highly heritable with 70% of the variation in risk attributed to genetics (Gharahkhani et al., 2023). To date, clinically, the causing factors contributing to glaucoma progression are still not well characterized. The most significant risk factor is elevated intraocular pressure (IOP). However, despite effective IOP-lowering therapies, visual impairment still progresses in a significant number of patients. Additionally, the most serious concern is that less than 50% of the general population has awareness of their glaucoma status. People who suffer from asymptomatic glaucoma may be significantly higher (Weinreb et al., 2014). Hence, evidence that identifies the risk factors for glaucoma is required urgently for the prevention of visual loss.

Recently, increasing epidemiological reports have illustrated that patients diagnosed with the most common psychiatric disorders such as depression (Jung et al., 2021; Wändell et al., 2022), insomnia (Sun et al., 2022), and schizophrenia (Meer et al., 2022) are more likely to have a higher risk of glaucoma, compared to the general population. It has been suggested that there might be a common underlying pathophysiology between psychiatric disorders and glaucoma, as both involve changes in vascular structures or neurological alterations (Liu et al., 2020). In a recent study, retinal nerve fiber layer thinning and neural cell loss in the ganglion cell layer were observed in the chronic unpredictable mild stress mouse model, indicating that psychological stress could induce glaucoma-like changes (Zhang et al., 2022). While some other epidemiological findings suggested that there was no association between depression and glaucoma (Rezapour et al., 2018; Vidal et al., 2021; Grant et al., 2021), which made this relationship contentious. Establishing a definitive etiological link may be challenging due to the presence of confounding factors and the potential for reverse causation in traditional epidemiological findings. More studies are needed to confirm the causal role of psychiatric disorders in glaucoma.

Mendelian randomization (MR) analysis, simulating the design of randomized control trials, uses genetic instrumental variables (single-nucleotide polymorphisms, SNPs) to assess the causal association between risk factors and outcomes, thereby excluding potential confounders from interfering (Emdin et al., 2017). So far, we found that the MR analysis of causality between psychiatric disorders and glaucoma was still unexplored. Hence, based on the data of genome-wide association studies (GWAS), this study aimed to reveal the causal association between three psychiatric disorders (depression, insomnia, and schizophrenia) and the risk of glaucoma through the two-sample MR analysis. Meanwhile, two main subtypes of glaucoma were explored and the causal effects in two different populations were evaluated respectively (the European and East Asian populations), aiming to contribute robust and novel insights to the field of the association between mental disorders and glaucoma.

## 2 Methods

### 2.1 Study design

**FIGURE 1:**
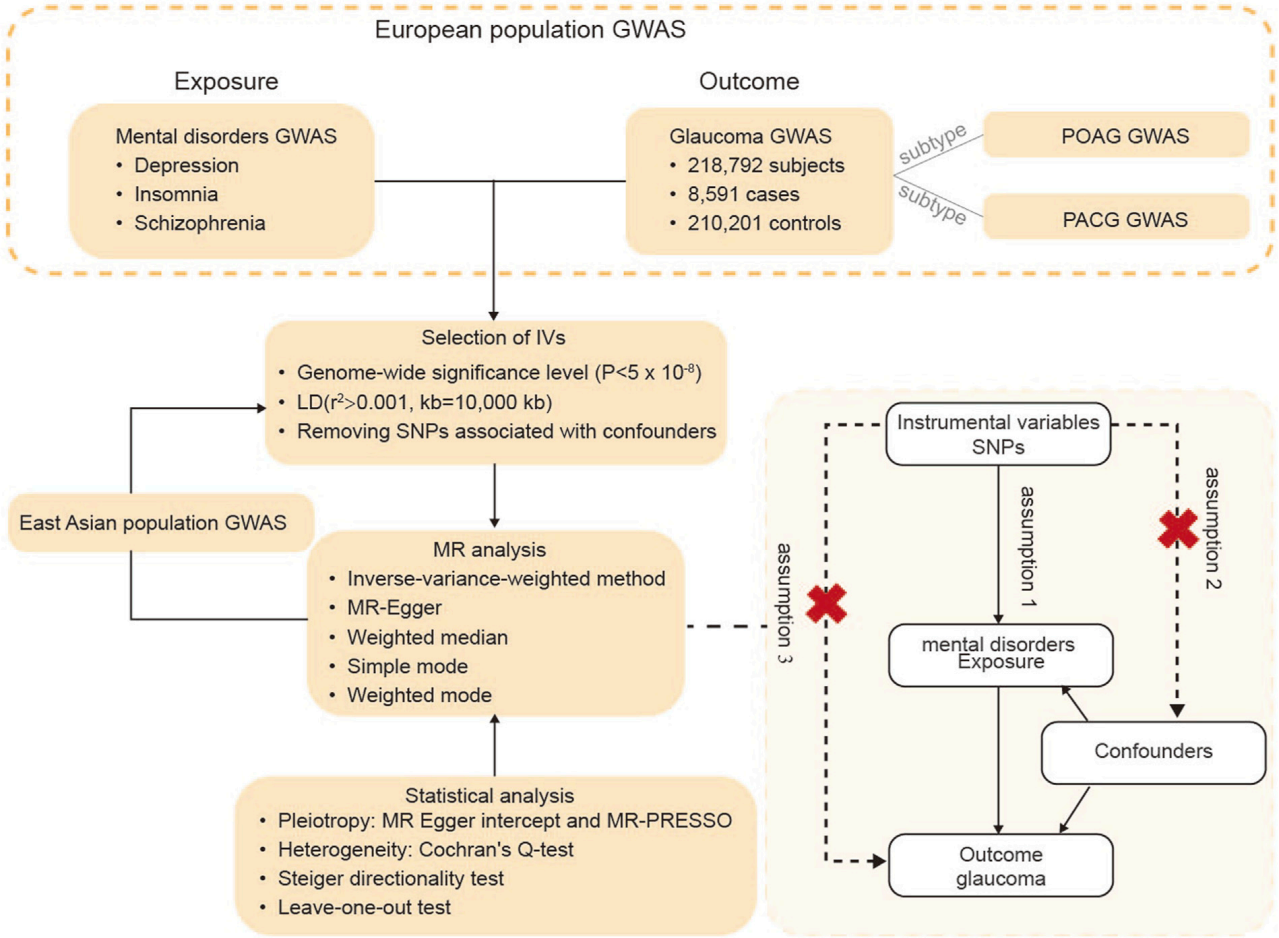
Diagram of the study design. The three key assumptions were as follows: (1) The genetic IVs must be associated with exposure (depression, insomnia, and schizophrenia); (2) IVs should not be associated with confounders; (3) IVs must influence glaucoma only via the exposure. Abbreviations: GWAS, genome-wide association studies; SNPs, single nucleotide polymorphisms; IVs, instrumental variables; LD, linkage disequilibrium; MR analysis, Mendelian randomization analysis; POAG, primary open-angle glaucoma; PACG, primary angle-closure glaucoma.

A flow diagram of the study design is presented in Figure 1. A two-sample MR analysis considering depression, insomnia, and schizophrenia as exposures and glaucoma as the outcome in the European population was conducted in the first step adhering to the three core assumptions (Burgess et al., 2019). Subgroup analysis of cases with POAG and primary angle-closure glaucoma (PACG) were also investigated. Sensitivity analysis was conducted to validate the robustness of the results. Finally, the analysis of individuals in East Asia was conducted for generalization and to provide an additional complement to the conclusion.

In order to adhere to the principle of minimizing duplication between exposure and outcome samples, we comprehensively searched the PubMed dataset and large publicly accessible GWAS data of European and East Asian ancestry samples to select the sample with rigor.

### 2.2 Data sources

#### 2.2.1 GWAS summary statistics in the European population

Summary statistics of depression in the European population were extracted from the largest European GWAS meta-analysis to date (170,756 cases and 329,443 controls) (Howard et al., 2019). As for insomnia, we obtained available summary data from UKB, which included 462,341 individuals. The GWAS data for schizophrenia were acquired from a dataset in the Psychiatric Genomics Consortium (PGC), which included a sample of 82,315 participants (Ripke et al., 2014).

The European GWAS data for glaucoma were identified from the FinnGen consortium, including three sets of genetic instruments (210,789 to 218,792 individuals): glaucoma, POAG, and PACG. Subtypes were included to further elucidate the causal relationship between genetically predicted psychiatric disorders and glaucoma. This study defined glaucoma by the International Classification of Diseases (ICD)-10: H40/H42.

#### 2.2.2 GWAS summary statistics in the East Asian population

Summary statistics of depression were derived from the hitherto most comprehensive and most up-to-date meta-analysis of depression GWAS among East Asians, comprising 98,502 individuals (12,588 cases and 85,914 controls) (Giannakopoulou et al., 2021). Data for insomnia published by UKB in 2020 that included a sample of 63,732 participants from East Asian populations were obtained from the Open GWAS database (https://gwas.mrcieu.ac.uk/). The East Asian statistics for schizophrenia were found from a large GWAS meta-analysis including 14,004 cases and 16,757 controls, which were open to download from the Psychiatric Genomics Consortium (Kurki et al., 2023).

The GWAS data for glaucoma in East Asian descent were derived from BioBank Japan (BBJ) (Ishigaki et al., 2020). BBJ is the largest East Asian biobank and includes more than 200000 Japanese people ranging in age from 20 to 89 years who were followed up between 2003 and 2018 (Ishigaki et al., 2020). The diagnosis of glaucoma was also defined by ICD-10: H40/H42. Summary-level GWAS data for specific glaucoma subtypes in the East Asian population was not included in the analysis because data was not publicly available. Detailed information of the data sources can be found in Table 1.

**TABLE 1:** Characteristics of data sources included in the MR analyses.

Traits	Population	Consortium	Sample size (case/controls)	PubMed ID/Open GWAS ID
Exposures				
Depression	European	UKB,PGC	500,199 (170,756/329,443)	30718901
Insomnia	European	UKB	462,341 (NA/NA)	ukb-b-3957
Schizophrenia	European	PGC	82,315 (35,476/46,839)	25056061
Depression	East Asian	UKB, CKB, etc.	98,502 (12,588/85,914)	34586374
Insomnia	East Asian	UKB	63,732 (2654/61,078)	ukb-e-1200_EAS
Schizophrenia	East Asian	PGC	30,761 (14,004/16,757)	35396580
Outcomes				
Glaucoma	European	Finn	218,792 (8,591/210,201)	finn-b-H7_GLAUCOMA
POAG	European	Finn	214,634 (4,433/210,201)	finn-b-H7_GLAUCPRIMOPEN
PACG	European	Finn	210,789 (588/210,201)	finn-b-H7_GLAUCPRIERM
Glaucoma	East Asian	BBJ	212,453 (5,761/206,692)	32514122

Note: POAG, Primary open-angle glaucoma; PACG, Primary angle-closure glaucoma; UK Biobank, the UK Biobank; PGC, Psychiatric Genomics Consortium; Finn, the FinnGen study; CKB, China Kadoorie Biobank; WHI, Women’s Health Initiative. All data was collected on 15 May 2023.

### 2.3 Selection of genetic instrumental variables

In this study, we employed criteria as follows to select the instrumental variables (IVs) (Burgess et al., 2019): (1) Firstly, all SNPs selected as instrumental variables were correlated with the corresponding exposure at a genome-wide significance (*p* < 5 × 10^−8^). As for IVs in the East Asian population, since the limitation of sample size, we adopted *p* < 5 × 10^−6^ as the threshold as recommended in previous research (Burgess et al., 2013). (2) The clumping process was executed to ensure that all the SNPs were not in linkage disequilibrium (LD) (r^2^ > 0.001, kb = 10,000) with the clump data function using the 1000 Genomes Project as the reference panel (Auton et al., 2015). (3) We used the PhenoScanner database (http://www.phenoscanner.medschl.cam.ac.uk/, accessed on 5 July 2023) to rule out SNPs related to confounding factors. (4) SNPs not available in the outcome dataset would also be excluded. (5) Genetic variables of palindromic and incompatible alleles were removed when harmonizing. Finally, F statistics of each SNP were calculated to avoid bias from weak instruments using the formula: $F={BETA}^{2}/{SE}^{2}$ , in which *BETA* represents the estimated effect size of allele and *SE* is the estimated standard error of *BETA* (Bowden et al., 2016b).

Herein, these SNPs were compliant with the correlation, independence, and statistical intensity requirements of instrumental variables.

### 2.4 Two sample MR analysis

For MR analysis, the inverse variance weighted (IVW) model which assumed all the IVs were valid was adopted as the main causal evaluation method (Burgess and Thompson, 2017). Different models of IVW were utilized based on the results of heterogeneity test. When the heterogeneity was large (*p* > 0.05), a random effects model would be applied to combine the effects. On the contrary, a fixed effects model would be used. Additionally, we applied MR-Egger, weighted median, and weighted mode as complementary methods. The MR-Egger regression method can also provide robust estimates when horizontal pleiotropy exists (Bowden et al., 2016b). The weighted median method can provide consistent effect estimates when up to 50% of the information comes from invalid instrumental variables (Bowden et al., 2016a). The weighted mode method detects a causal effect smaller compared with the IVW and weighted median methods, with sample size requirements typically smaller than those available from GWAS consortia (Hartwig et al., 2017). Scatter plots were used to visualize analysis results.

### 2.5 Robust analysis

To further confirm the robustness of the analysis result, heterogeneity was assessed through Cochran’s Q test in the IVW approach. We used funnel plots to visualize potential bias, where a symmetrical funnel suggests little bias. We settled MR-Egger regression to examine the existence of horizontal pleiotropy (Burgess and Thompson, 2017) and adopted the MR-pleiotropy residual sum and outlier method (MR-PRESSO) test as a supplement (Verbanck et al., 2018). When the MR PRESSO test showed outliers, the MR PRESSO test examined whether there was significant distortion in the results after removing the outliers. On top of that, a leave-one-out analysis was performed to estimate the stability of the findings, which successively excluded one SNP at a time to check whether the result was biased or driven by a single SNP (Emdin et al., 2017). Furthermore, we performed the MR Steiger directionality test to confirm the causality direction between psychiatric disorders and glaucoma (Hemani et al., 2017).

All statistical analyses were performed with R software 4.3.0 using the “TwoSampleMR” package (version 0.5.7) and “MR PRESSO” (version 1.0) package. The Bonferroni-corrected *p*-value<0.004 (0.05/12) adjusted for multiple testing was considered statistically significant.

## 3 Results

### 3.1 The SNPs used as instrumental variables

We obtained 80 SNPs in depression, 42 SNPs in insomnia, and 83 SNPs in schizophrenia among the European population, which met the generally accepted genome-wide significance threshold (*p* < 5 × 10^−8^, r^2^ < 0.001, kb = 10,000) for exposure. Subsequently, 26 SNPs in depression, 9 SNPs in insomnia, and 45 SNPs in schizophrenia were found available at the significant level (*p* < 5 × 10^−6^, r^2^ < 0.001, kb = 10,000) among East Asian GWAS. Some SNPs significantly correlated with confounding factors such as hypertension (Shukla et al., 2020), diabetes (Choi et al., 2020), body mass index (Leske et al., 1995), waist circumference (Yuan et al., 2022), platelet count (Ma et al., 2019), basophil cell count (Song et al., 2023), lymphocyte count (Yang et al., 2001) were eliminated. The detailed information about eliminated SNPs is listed in Supplementary Table S1. All selected SNPs had F-statistics larger than threshold 10 (ranging from 16.0 to 199.3), indicating no weak instrument bias existed. Detailed information about the used genetic instruments of exposures is presented in Supplementary Tables S2–S4. Scatter plots of different exposures in this study are presented in Supplementary Figures S1–S3.

### 3.2 MR analysis results in the European population

#### 3.2.1 Depression

**FIGURE 2:**
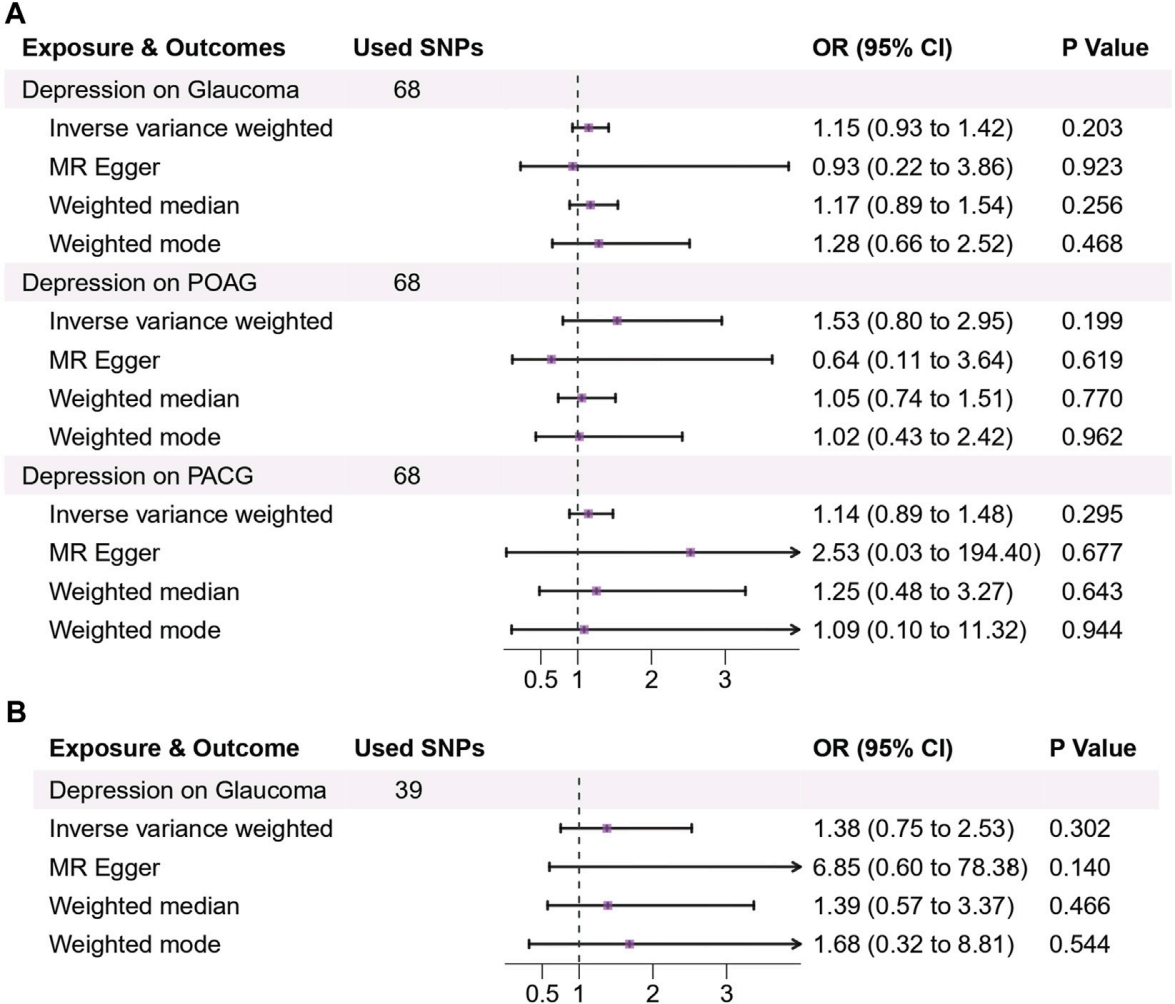
Genetic causal associations between depression and the risk of glaucoma in the European **(A)** and East Asian **(B)** populations. **(A)** MR estimates of genetically predicted risk of depression on glaucoma, primary open-angle glaucoma, and primary angle-closure glaucoma in the European population. **(B)** MR estimates of genetically predicted risk of depression on glaucoma in the East Asian population. The inverse variance weighted method is considered the main method. Abbreviations: SNPs, single nucleotide polymorphisms; OR, odds ratio; 95% CI, 95% confidence interval.

In the primary IVW results, depression showed a non-causal association with the risk of glaucoma (OR: 1.15, 95% CI: 0.93–1.42, *p* = 0.20) and glaucoma subtypes (POAG: OR: 1.53, 95% CI: 0.80–2.95, *p* = 0.20; PACG: OR:1.14, 95% CI: 0.89–1.48, *p* = 0.30). The other methods including MR-Egger, weighted median, and weighted mode were consistent with IVW results, indicating that depression had no MR association with the risk of glaucoma through either glaucoma or specific subtypes (POAG and PACG), with all *p*-values greater than 0.05 (Figure 2).

#### 3.2.2 Insomnia

**FIGURE 3:**
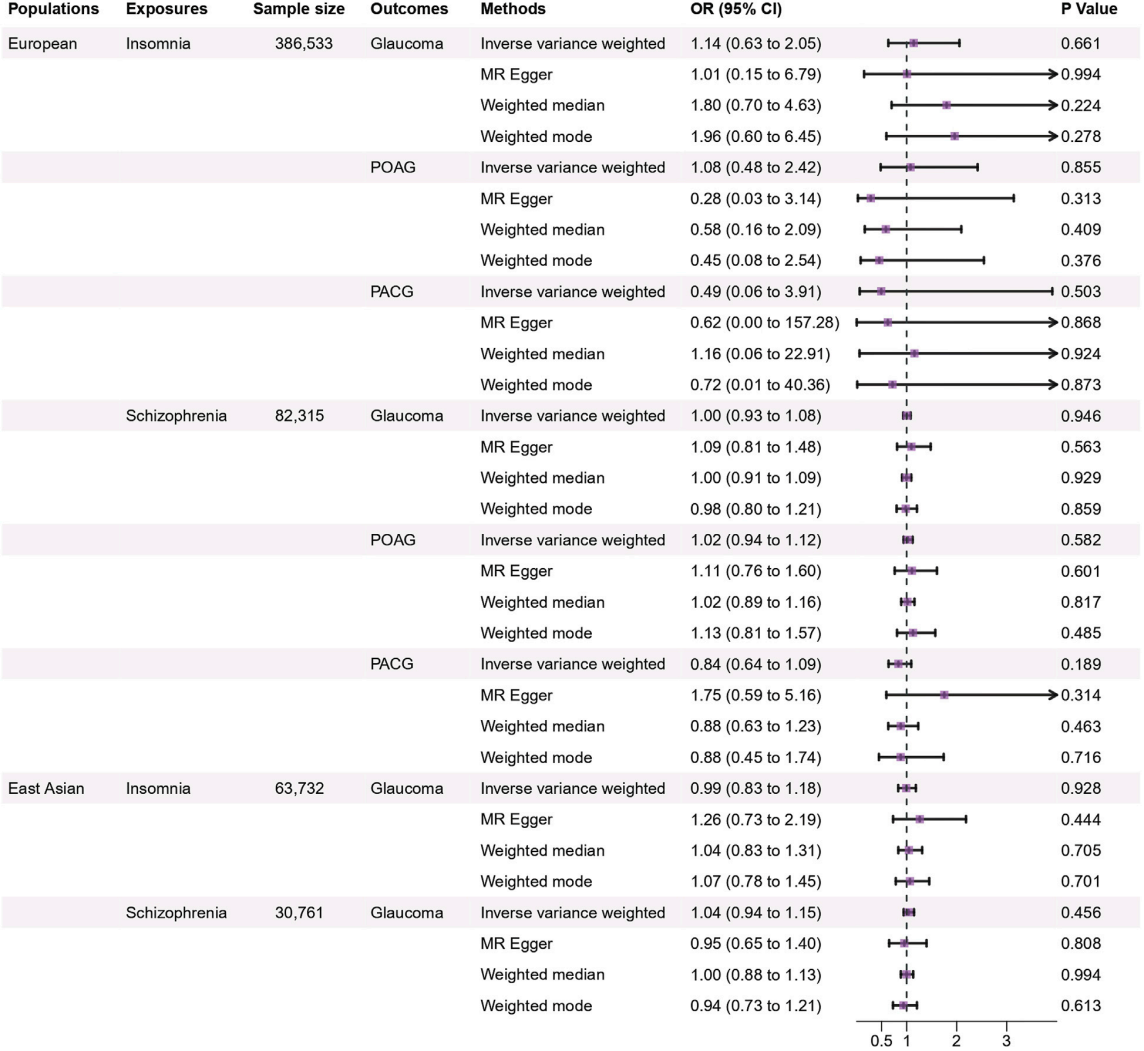
Genetic causal associations of insomnia and schizophrenia on the risk of glaucoma in the European and East Asian populations. The inverse variance weighted method is considered the main method. Abbreviations: SNPs, single nucleotide polymorphisms; OR, odds ratio; 95% CI, 95% confidence interval.

From the MR analyses between insomnia and glaucoma, the overall causality estimated by the IVW method indicated no potential causal relationship between insomnia and glaucoma (OR: 1.14, 95% CI: 0.63–2.05, *p* = 0.66). Same as POAG and PACG (OR: 1.08, 95% CI: 0.48–2.42, *p* = 0.86; OR: 0.49, 95% CI: 0.06–3.91, *p* = 0.50), no causal relationship between insomnia and glaucoma was detected. The MR Egger and weighted median results also showed no association between insomnia and glaucoma (Figure 3).

#### 3.2.3 Schizophrenia

**FIGURE 4:**
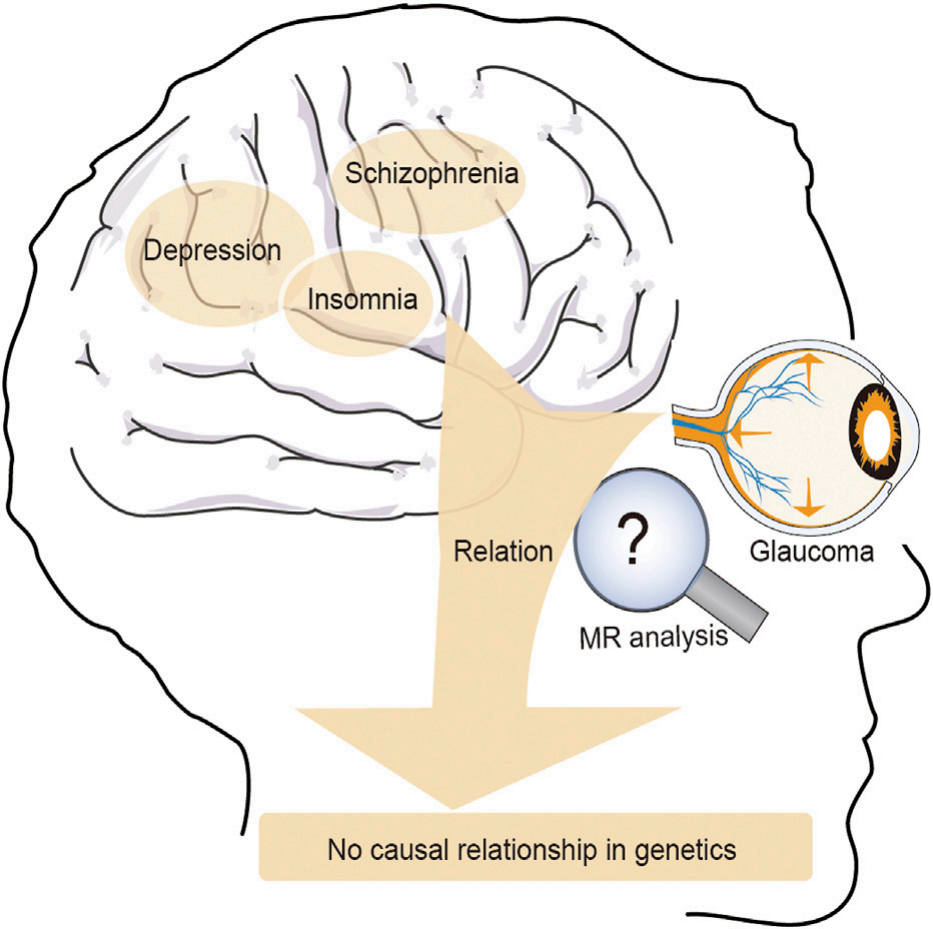
Genetic causal associations between psychiatric disorders and glaucoma.

MR analysis results of schizophrenia did not reveal that schizophrenia could increase the risk of glaucoma (IVW method: OR: 1.00, 95% CI: 0.93–1.08, *p* = 0.95). The similar results were found for POAG and PACG (IVW method: OR: 1.02, 95% CI: 0.94–1.12, *p* = 0.58; OR: 0.84, 95% CI: 0.64–1.09, *p* = 0.19). These results were corroborated by other methods, indicating that schizophrenia had no MR association with the risk of glaucoma and two subtypes (Figure 3). A brief cartoon describing the result is shown in Figure 4.

#### 3.2.4 MR analysis results in the East Asian population

Replicated analyses in the East Asian population validated the estimation in the European population (all *p* > 0.05). The primary IVW results showed that genetically predicted psychiatric disorders had no causal association with glaucoma (Depression: OR: 1.38, 95% CI: 0.75–2.53, *p* = 0.30; Insomnia: OR: 0.99, 95% CI: 0.83–1.18, *p* = 0.93; Schizophrenia: OR: 1.04, 95% CI: 0.94–1.15, *p* = 0.46), with similar causal estimates in direction in the European population. Consistency was obtained by corroborating with the other methods including MR-Egger, weighted median, and weighted mode (Figures 2, 3).

#### 3.2.5 Robustness of the MR analysis

Potential SNP heterogeneity evaluated by the IVW method was observed in instrumental variables of depression and schizophrenia in the European population (Cochran’s Q *p* < 0.05), in which the random IVW effects model was adopted. In other exposures we analyzed, no evidence of heterogeneity was detected as indicated by the results of Cochran’s Q-test (all *p*-values>0.05). Additionally, the funnel plots that visualized the heterogeneity were presented (Supplementary Figures S4–S6). Regarding pleiotropy, in all analyses we studied in two different populations, the Egger intercept quantified by the MR-Egger regression method did not differ significantly from zero (*p* > 0.05), which indicated no evidence of horizontal pleiotropic effect. Results remained consistent with IVW results after excluding outliers SNP through the MR-PRESSO distortion test, reconfirming the absence of horizontal pleiotropy (*p* > 0.05) (Supplementary Table S5). The leave-one-out plots demonstrated that the exclusion of any single SNP used in the analysis had no significant impact on the causal association and so draw up the reliability of the causal effect estimates (Supplementary Figures S7–S9). Besides, the results of the MR Steiger directionality test supported our hypothesis regarding the potential causal direction between psychiatric disorders and glaucoma (*p* < 0.001) (Supplementary Table S5).

## 4 Discussion

This study applied a two-sample MR method to study the causal relationships among three highly related psychiatric disorders and glaucoma, as well as two glaucoma subtypes (POAG and PACG) using large publicly available GWAS summary statistics. Our findings suggested a non-genetic association between depression and glaucoma utilizing the biggest GWAS to date. Meanwhile, there was no evidence indicating that genetically predicted insomnia and schizophrenia were causally related to the risk of glaucoma. Replicated analyses in the East Asian population showed consistency with the results among the European population, with similar causal estimates in amplitude and the same causal estimates in direction. These findings gave us another vision that increasing psychiatric disorders in glaucoma patients observed in previous studies may be more likely to be attributed to modifiable factors rather than inheritable factors.

It is widely known that patients with glaucoma who have irreversible vision impairment often experience continuous mental stress. The worries of losing independence trigger fear with secondary consequences such as depression. A meta-analysis indicated a higher prevalence and severity of depression, anxiety, and sleep disorders in patients with glaucoma (Groff et al., 2023). While prolonged mental stress and psychiatric disorders may not only be a result but also a possible cause (Sabel and Lehnigk, 2021) and a risk factor of glaucoma progression (Shin et al., 2021). Recently, increasing reports from observational studies have found that psychiatric disorders may likely to associated with a higher risk of glaucoma. A study screened all living individuals with specified psychiatric disorders in the years 2010–2019 who resided in Stockholm County, indicating that the risk of POAG was increased in women with depression (Wändell P. E. et al., 2022). A prospective cohort study in the UK investigated the link between sleep behavior and pattern with the risk of glaucoma and found that individuals with insomnia had an excess risk of any glaucoma (Hazard ratio:1.13, 95% CI:1.06–1.20) (Sun et al., 2022). A hospital-based comparative study that comprised 180 patients diagnosed with varying degrees of severity of POAG found that the glaucoma patients showed evidence of poor mental health with 39 (21.7%) of them depressed compared to controls (*p* < 0.001) (Ubochi et al., 2020). Liu et al. observed that glaucoma suspect (OR: 1.88, 95% CI: 1.01–3.49) and OAG (OR: 2.19, 95% CI: 1.13–4.26) showed significant associations with schizophrenia (Liu et al., 2020). However, a prospective cohort research revealed that there was no link between depression and glaucoma, which was inconsistent with findings forementioned (Vidal et al., 2021). Also, a 3-year longitudinal study consisting of 30,097 individuals aged 45–85 years did not find an association between glaucoma and depression (Grant et al., 2021). These contentious findings may be influenced by indissoluble or unidentified risk factors in observational studies.

Several factors may interfere the judgment of causality. Firstly, the causative relationship might be overestimated, considering that psychiatric disorders could be secondary to the glaucoma diagnosis even secondary to the use of antiglaucoma medication. Secondly, some antidepressants such as topiramate (Kocamaz and Karadag, 2019), aripiprazole (Shen et al., 2018), milnacipran (Keks et al., 2018), and duloxetine (Mahmut et al., 2017) have potential eye side effects, such as acute onset angle-closure glaucoma. Chen et al. (2016) found that patients using selective serotonin reuptake inhibitors (SSRIs) have a 5.80-fold increased risk of angle-closure glaucoma in a week. Side effects of antidepressants may affect the accuracy of the findings. Besides, gender was found to have a significant effect on the mental health of glaucoma patients. An institution-based cross-sectional study conducted on 495 glaucoma patients indicated that the female sex (95% CI: 1.66–8.62) (*p* = 0.001) was significantly associated with increased levels of common mental disorders in glaucoma patients (Tilahun et al., 2021). It is in harmony with the study of Lim et al. (2016), reporting higher depression and anxiety in females than in male glaucoma patients. While it is at variance with the report by Ubochi et al. (2020) which revealed that males had higher depression and anxiety scores than females. As glaucoma and depression are known to be more common among female individuals, the ratio of female patients and the differences in methodology may interfere with the outcome (El-Mogy et al., 2014). In this study, the ratio of female participants is not noted because the ratio cannot be obtained directly from GWAS summary statistics and original articles. Lastly, glaucoma is a group of optic neuropathies that contains different subtypes, overall assessment criteria may misjudge a certain link, and primary glaucoma and secondary glaucoma should be taken into account separately in the investigation. Thus, we employed MR analysis that could exclude the influence of external confounding factors on this contentious relationship so as more likely to draw a reliable genetic causal conclusion. To summarize, rather than the diseases themselves, our non-causal findings indicated that the link between psychiatric problems and glaucoma may emerge through other manageable pathways. This non-causal conclusion has important clinical significance for ophthalmology as further understanding of psychological mechanisms would have to be considered more in the treatment of glaucoma than the development of glaucoma.

This study has several prominent advantages. Firstly, the MR method is the closest approximation to the randomized controlled trial which emulates the random allocation procedure. Theoretically, the influence of external confounding factors can be excluded using MR method, making MR study immune to some limitations of conventional observational studies. Secondly, in our study, the latest and largest publicly accessible GWAS data and strict SNP filtering criteria were used to provide solid evidence for the results. A variety of analytical methods are utilized. Several sensitivity tests were engaged to ensure the robustness of the results. The MR-Egger analysis and MR-PRESSO test suggested no horizontal pleiotropy. Lastly, we performed analysis across two different ancestries (the European and the East Asian populations) and explored subgroup effects (POAG and PACG), which intensified generalizability and validated the adaptation of our results more comprehensively.

Several limitations should be taken into consideration. First and foremost, potential pleiotropy could not be completely voided through the current finite test methods. Secondly, in order to adhere to the principle of minimizing duplication between exposure and outcome samples, the data sources we used are from different institutions. The difference in data collection criteria and diagnosis coding across institutions might affect the estimation of results, especially institutions from different populations. Thirdly, although two different populations had been enrolled in our analysis, it was restricted in other populations due to potential inter-ethnic genetic differences. Additionally, the lack of publicly available GWAS on glaucoma subtypes and other psychiatric disorders like anxiety, disorder, and bipolar disorder in the East Asian population precluded us from exploring the effect in MR analysis. Thus, there is likely to be a need for big sample GWAS and new loci studies of psychiatric disorders and glaucoma across different ancestries.

## 5 Conclusion

In conclusion, our MR analysis results did not support that genetically predicted psychiatric disorders (including depression, insomnia, and schizophrenia) have any causal effect on the risk of glaucoma, indicating that increased psychiatric disorders in glaucoma patients were more likely not inheritable but modifiable. The findings address an importance of keeping an eye on the mental health of eye disorders and necessitate further research to fully understand this relationship in the future.

## Data Availability

The original contributions presented in the study are included in the article/Supplementary Material, further inquiries can be directed to the corresponding author.
