# Supplementary material for: Mendelian randomization study shows no causal relationship between psychiatric disorders and glaucoma in European and East Asian populations
Source: Front Genet. 2024 Mar 7;15:1349860. doi: 10.3389/fgene.2024.1349860 (PMC10954835; doi:10.3389/fgene.2024.1349860)
Supplement: Supplementary file 2 [file Image1.pdf]

Supplementary Figures

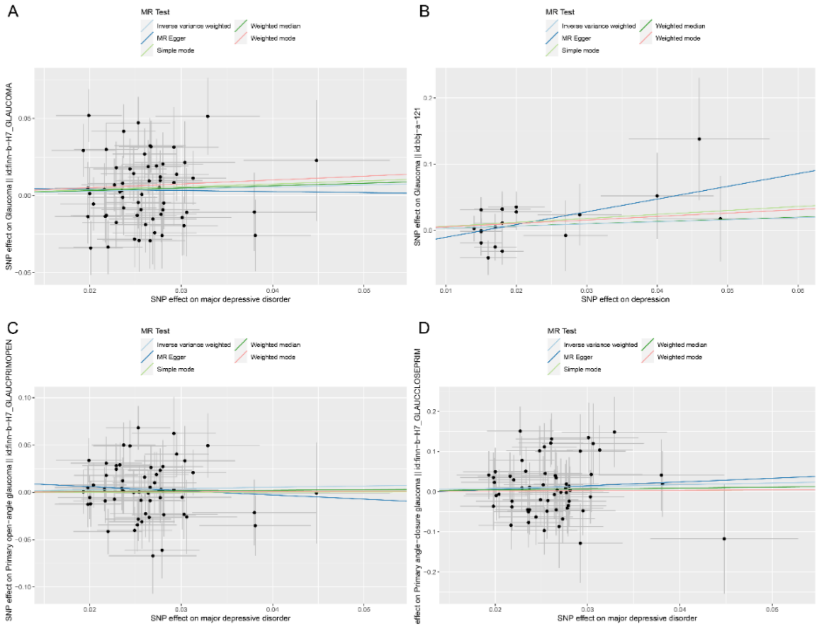

Supplementary Figure S1. Scatter plots of estimates for the association of depression on (A) Glaucoma in European population, (B) Glaucoma in East Asian population, (C) POAG in European population, (D) PACG in European population. Abbreviations: MR: mendelian randomization; SNPs: Single Nucleotide Polymorphisms; POAG: primary open-angle glaucoma; PACG: primary angle-closure glaucoma.

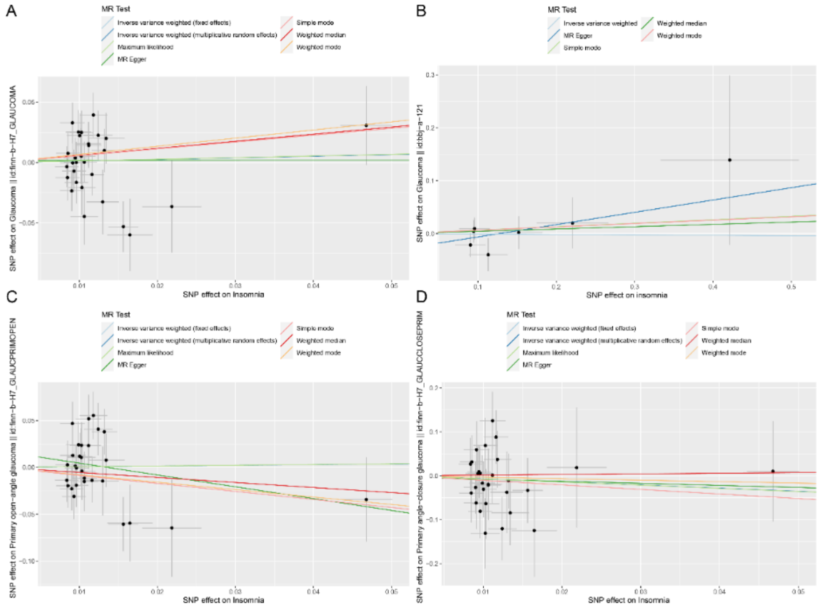

Supplementary Figure S2. Scatter plots of estimates for the association of insomnia on (A) Glaucoma in European population, (B) Glaucoma in East Asian population, (C) POAG in European population, (D) PACG in European population. Abbreviations: MR: mendelian randomization; SNPs:

Single Nucleotide Polymorphisms; POAG: primary open-angle glaucoma; PACG: primary angle-closure glaucoma

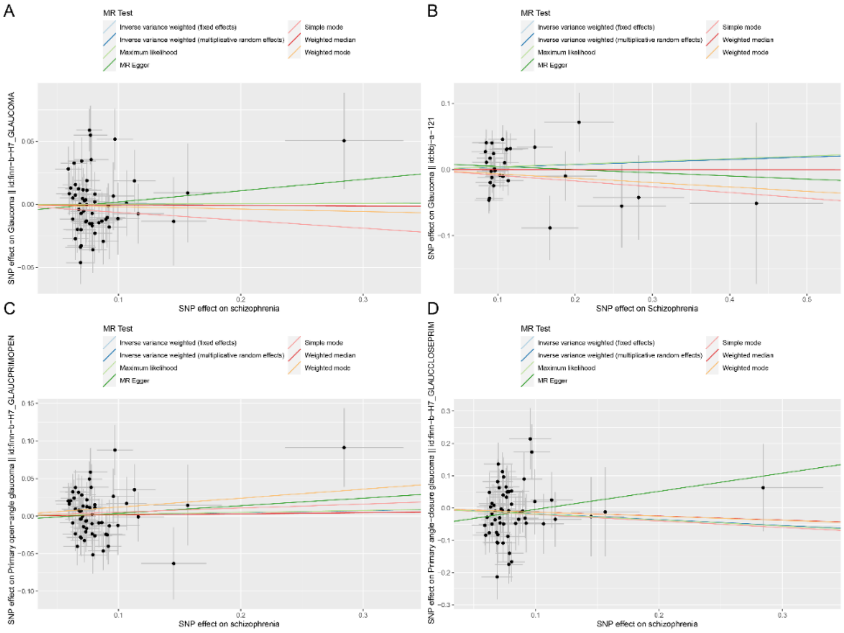

Supplementary Figure S3. Scatter plots of estimates for the association of schizophrenia on (A) Glaucoma in European population, (B) Glaucoma in East Asian population, (C) POAG in European population, (D) PACG in European population. Abbreviations: MR: mendelian randomization; SNPs: Single Nucleotide Polymorphisms; POAG: primary open-angle glaucoma; PACG: primary angle-closure glaucoma.

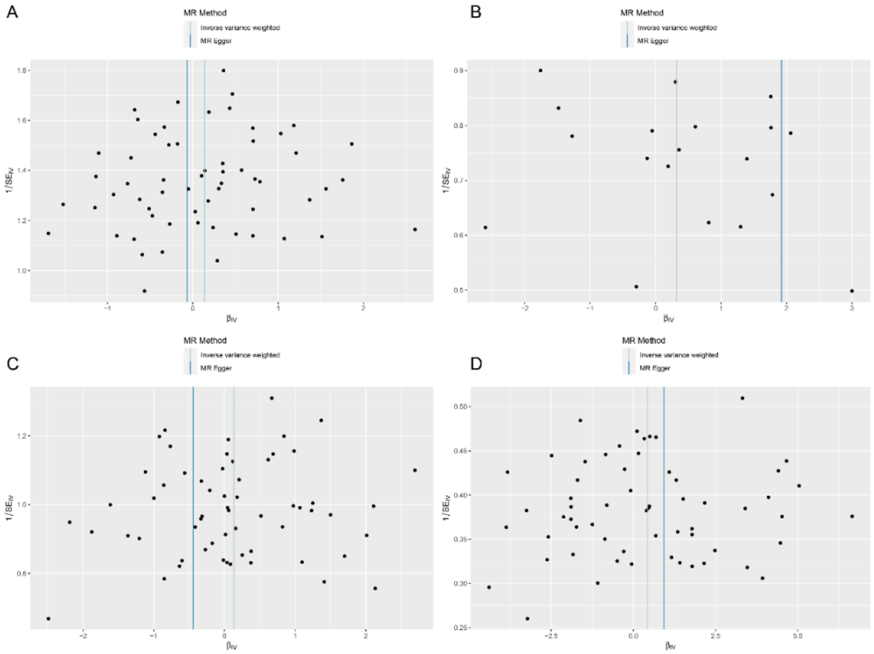

Supplementary Figure S4. Funnel plots of estimates for the association of depression on (A) Glaucoma in European population, (B) Glaucoma in East Asian population, (C) POAG in European population, (D) PACG in European population. Abbreviations: MR: mendelian randomization.

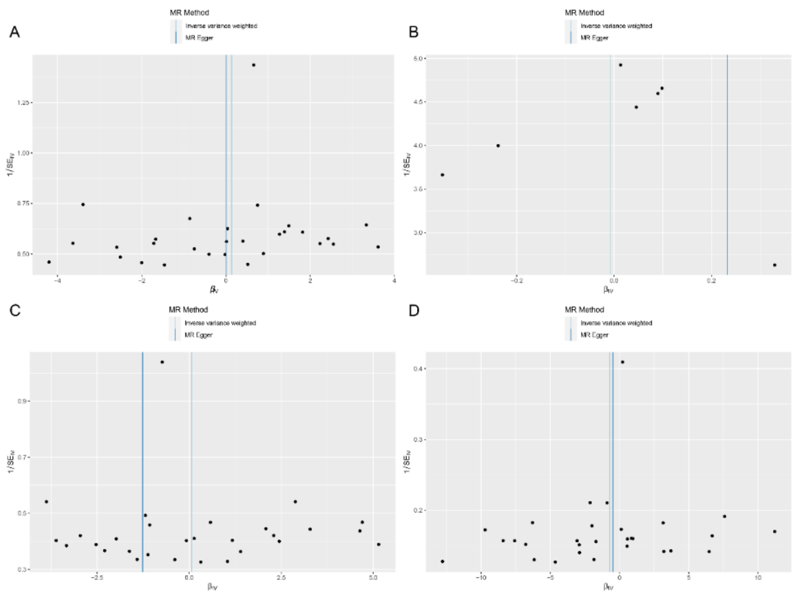

Supplementary Figure S5. Funnel plots of estimates for the association of insomnia on (A) Glaucoma in European population, (B) Glaucoma in East Asian population, (C) POAG in European population, (D) PACG in European population. Abbreviations: MR: mendelian randomization.

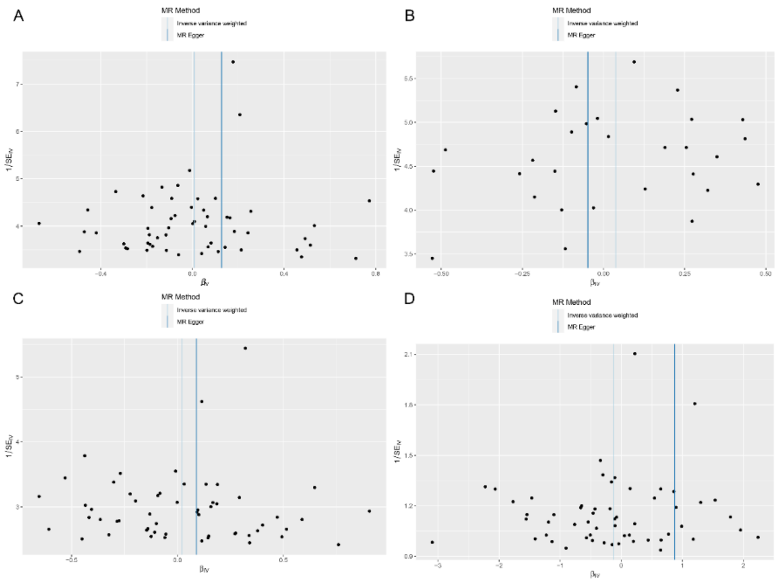

Supplementary Figure S6. Funnel plots of estimates for the association of schizophrenia on (A) Glaucoma in European population, (B) Glaucoma in East Asian population, (C) POAG in European population, (D) PACG in European population. Abbreviations: MR: mendelian randomization.

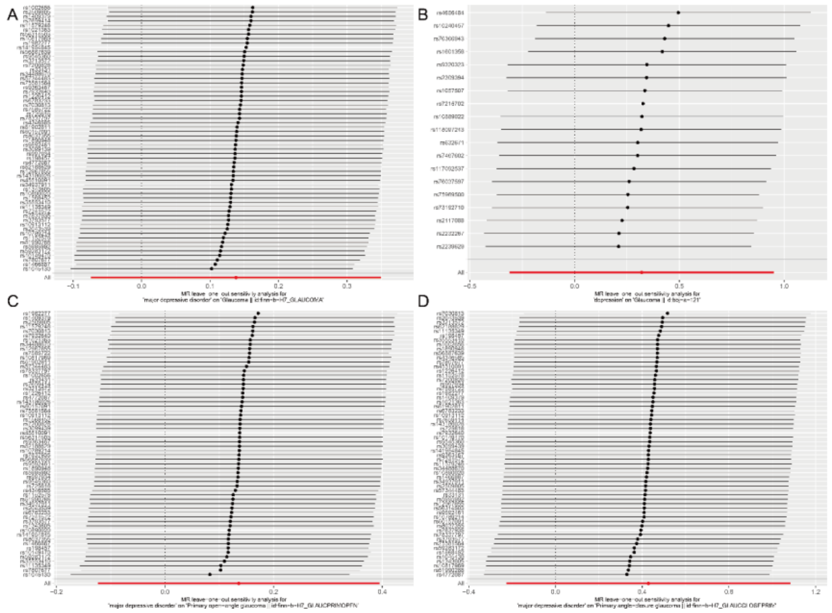

Supplementary Figure S7. Leave-one-out results for depression on (A) Glaucoma in European population, (B) Glaucoma in East Asian population, (C) POAG in European population, (D) PACG in European population. Abbreviations: MR: mendelian randomization.

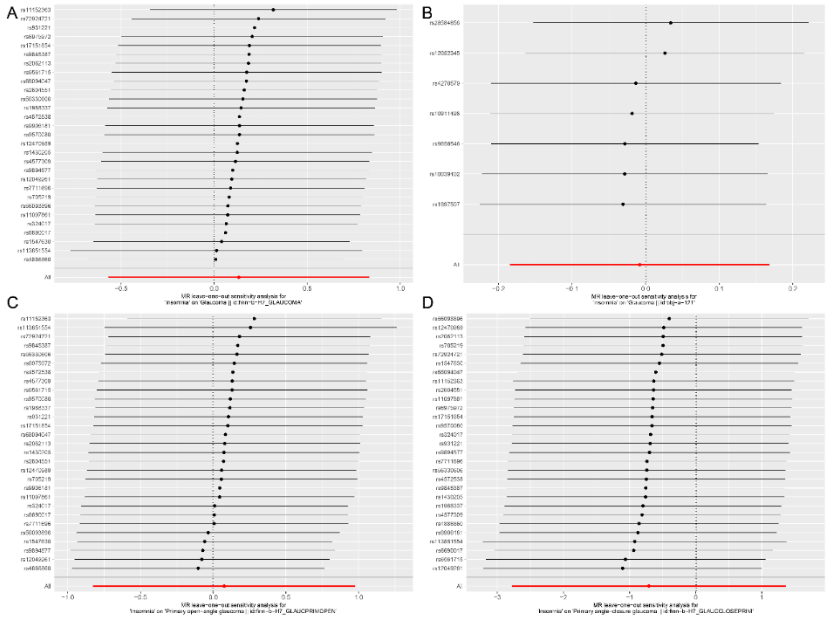

Supplementary Figure S8. Leave-one-out results for insomnia on (A) Glaucoma in European population, (B) Glaucoma in East Asian population, (C) POAG in European population, (D) PACG in European population. Abbreviations: MR: mendelian randomization.

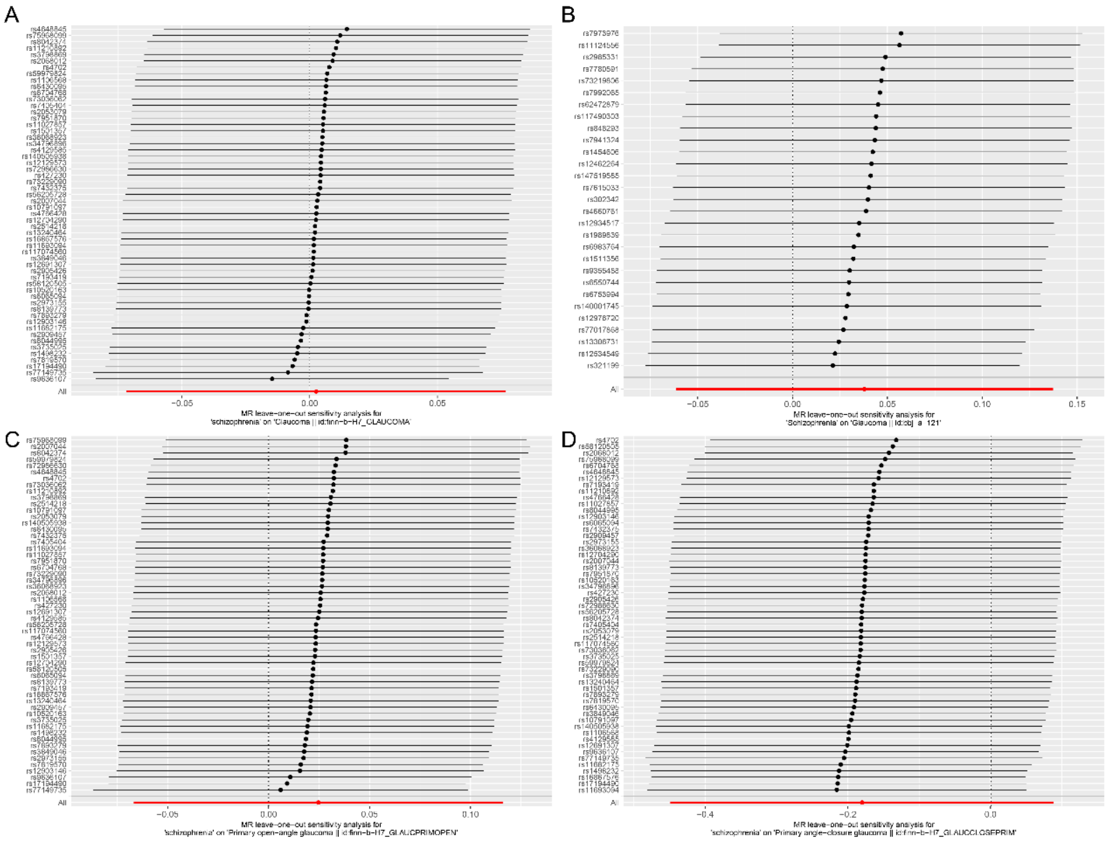

Supplementary Figure S9. Leave-one-out results for schizophrenia on (A) Glaucoma in European population, (B) Glaucoma in East Asian population, (C) POAG in European population, (D) PACG in European population. Abbreviations: MR: mendelian randomization.
